# Transcription factors and molecular epigenetic marks underlying EpCAM overexpression in ovarian cancer

**DOI:** 10.1038/bjc.2011.231

**Published:** 2011-06-21

**Authors:** B T F van der Gun, M L de Groote, H G Kazemier, A J Arendzen, P Terpstra, M H J Ruiters, P M J McLaughlin, M G Rots

**Affiliations:** 1 Epigenetic Editing, Department of Pathology and Medical Biology, University Medical Centre Groningen, University of Groningen, Hanzeplein 1, 9713 GZ Groningen, The Netherlands; 2 Epidemiology, Department of Genetic Epidemiology and Bioinformatics, University Medical Centre Groningen, University of Groningen, Hanzeplein 1, 9713 GZ Groningen, The Netherlands; 3 Synvolux Therapeutics Inc., LJ. Zielstraweg 1, 9713 GX Groningen, The Netherlands

**Keywords:** ovarian cancer, EpCAM, DNA methylation, histone modifications, transcription factors

## Abstract

**Background:**

The epithelial cell adhesion molecule (EpCAM) is overexpressed on carcinomas, and its downregulation inhibits the oncogenic potential of multiple tumour types. Here, we investigated underlying mechanisms of epcam overexpression in ovarian carcinoma.

**Methods:**

Expression of EpCAM and DNA methylation (bisulphite sequencing) was determined for ovarian cancer cell lines. The association of histone modifications and 16 transcription factors with the *epcam* promoter was analysed by chromatin immunoprecipitation. Treatment with 5-Aza-2′-deoxycytidine (5-AZAC) was used to induce EpCAM expression.

**Results:**

Expression of EpCAM was correlated with DNA methylation and histone modifications. Treatment with 5-AZAC induced EpCAM expression in negative cells. Ten transcription factors were associated with the *epcam* gene in EpCAM expressing cells, but not in EpCAM-negative cells. Methylation of an Sp1 probe inhibited the binding of nuclear extract proteins in electromobility shift assays; such DNA methylation sensitivity was not observed for an NF-*κ*B probe.

**Conclusion:**

This study provides insights in transcriptional regulation of *epcam* in ovarian cancer. Epigenetic parameters associated with EpCAM overexpression are potentially reversible, allowing novel strategies for sustained silencing of EpCAM expression.

The epithelial cell adhesion molecule (EpCAM; CD326) is a transmembrane glycoprotein, highly overexpressed on most carcinomas. Recently, EpCAM also gained interest as a signal transducer (Maetzel *et al*, 2009) and as a marker of cancer-initiating cells (Visvader and Lindeman, 2008). The role of EpCAM in the development of cancer and in tumour progression is dependent on the tumour type as recently reviewed by us (van der Gun *et al*, 2010b). For example, in breast cancer, high EpCAM expression correlates with poor prognosis (Spizzo *et al*, 2004), and downregulation of EpCAM has been shown to decrease the oncogenic potential (Osta *et al*, 2004). In contrast, high EpCAM expression in, for example, primary renal cell carcinomas is associated with improved patient survival (Seligson *et al*, 2004; Klatte *et al*, 2009). In other types of carcinoma such as ovarian cancer, the role of EpCAM is not clear and contradictory results have been reported.

In normal ovary and benign ovarian tumours, EpCAM expression is lower compared with malignant ovarian tumours (Kim *et al*, 2003). Numerous studies confirmed the EpCAM overexpression in ovarian carcinomas (Heinzelmann-Schwarz *et al*, 2004; Spizzo *et al*, 2006; Kobel *et al*, 2008), turning EpCAM into a well-established ovarian tumour marker (Spizzo *et al*, 2011). The role of EpCAM in ovarian tumour progression, however, is unclear; one study reported that FIGO stage III/IV showed lower EpCAM expression than stage I (Kim *et al*, 2003), while in another study, FIGO stage III/IV showed higher EpCAM expression than stage I/II disease (Heinzelmann-Schwarz *et al*, 2004). Importantly, metastatic and recurrent tumours were found to express significantly higher levels of EpCAM protein when compared with primary ovarian carcinomas (Bellone *et al*, 2009). Despite some contradictory results, the observations suggest a promoting rather than a protecting role for EpCAM in ovarian cancer. This promoting role is further confirmed for patients with stage III/IV disease, for whom EpCAM overexpression was shown to correlate significantly with decreased overall survival (Spizzo *et al*, 2006).

Besides its possible prognostic role in ovarian cancer, EpCAM is used as a therapeutic immunotarget for the treatment of malignant ascites. For example, catumaxomab is a trifunctional monoclonal antibody (anti-EpCAM × anti-CD3) approved to treat ovarian cancer patients with malignant ascites (Bokemeyer, 2010). Recently, it has been reported that catumaxomab treatment might also have an effect on tumour cells in blood of ovarian cancer patients (Wimberger *et al*, 2009). Similarly, the human monoclonal antibody MT201 could effectively eliminate malignant cells in metastic tumour specimens from patients with ovarian cancer (Xiang *et al*, 2003).

For various tumour types, EpCAM overexpression has been associated with DNA hypomethylation of the promoter and treatment of EpCAM-negative cells with a DNA methylation inhibitor induced EpCAM expression (Spizzo *et al*, 2007; Tai *et al*, 2007; van der Gun *et al*, 2008). Alternatively, we also demonstrated that endogenous EpCAM expression can be actively downregulated by nuclear delivery of a DNA methyltransferase (van der Gun *et al*, 2008). Here, we investigate epigenetic mechanisms and transcription factors underlying the overexpression of EpCAM in ovarian cancer. Unlike genetic mutations, epigenetic mutations are reversible; a better understanding of the regulation of EpCAM gene expression may thus provide new opportunities for cancer therapy, based on reversing epigenetic marks.

## Materials and methods

### Cell culture and 5-AZAC treatment

Ovarian cancer cell lines (H134S, SKOV3, CaOV3, OVCAR3) were cultured in DMEM (BioWhittaker, Walkersville, MD, USA) and A2780 in RPMI-1640 (BioWhittaker) with 50 *μ*g ml^−1^ gentamicin sulphate, 2 mM L-glutamine and 10% FBS. Culture medium of A2780 contained also 1 mM Na-pyruvaat and 0.05 mM *β*-mercaptoethanol. For DNA methylation inhibition studies, H134S and A2780 were cultured in their appropriate media with a final concentration of 5 *μ*M 5-Aza-2′-deoxycytidine (5-AZAC; Sigma, St Louis, MO, USA). Everyday, freshly prepared 5-AZAC was added, and after 3 days, cells were harvested for extraction of protein and mRNA.

### EpCAM protein expression

EpCAM protein was detected by mouse Mab MOC31 hybridoma supernatant, followed by R*α*M-F(ab)_2_-FITC (DAKO, Glostrup, Denmark) or mouse CD326-APC (Biolegend, Uithoorn, the Netherlands). The mean fluorescence intensity (MFI) was measured on a Calibur flow cytometer (Beckton Dickinson Biosciences, San Jose, CA, USA).

### Quantitative gene expression analysis by real-time RT–PCR

RNA was isolated using Rneasy Mini Kit (Qiagen, Venlo, The Netherlands); 1 *μ*g was reverse-transcribed (RevertAid cDNA Synthesis Kit (Fermentas, Leon-Rot, Germany). The Q-PCR analysis was performed (ABIPrism 7900HT, Applied Biosystems, Nieuwekerk, the Netherlands) for EpCAM (Hs00158980_m1, Applied Biosystems) and GAPDH (F5′-CCACATCGCTCAGACACCAT-3′, R5′-GCGCCCAATACGACCAAAT-3′, probe: CGTTGACTCCGACCTTCACCTTCCC (Eurogentec, Maastricht, the Netherlands)) in triplicate. Relative gene expression levels were calculated based on the comparative cycle treshold (*C*_t_) method (Δ*C*_t_=*C*_t_ EpCAM−*C*_t_ GAPDH). To compare EpCAM expression of different samples, the differences in Δ*C*_t_ of individual samples (ΔΔ*C*_t_) were used (A2780 was set at 1).

### DNA methylation analysis

**Figure 1:**
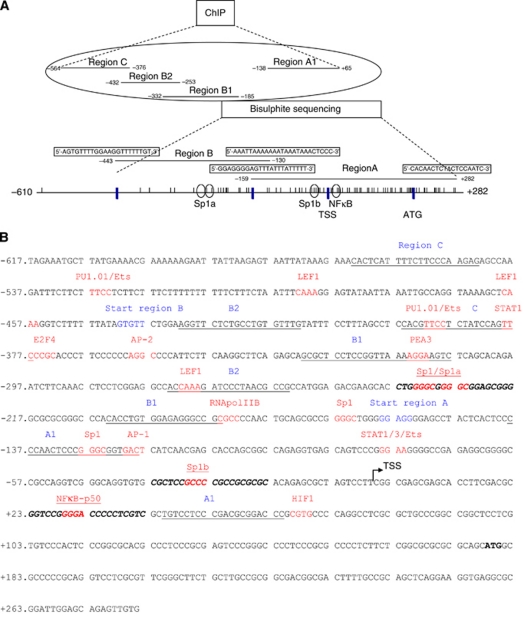
Part of the *epcam* gene under investigation. (**A**) Schematic overview: nucleotide position −610 to +282 relative to the transcription starting site (TSS); the ATG start codon is shown; CpGs are depicted by vertical bars. Regions A and B were analysed for DNA methylation, region C, B2 and A1 for histone modifications, and region B1 and A1 for transcription factors. Open circles represent putative Sp1 and NF-*κ*B binding sites. (**B**) Nucleotide positions −617 to +282 relative to the TSS are shown; the ATG start codon is depicted in bold. The start site of regions A and B is indicated in blue and PCR primers are underlined. Putative transcription factor binding sites analysed by *in silico* analysis (Genomatix, MatInspector version 7.7.3.1) are indicated in red. Probes for EMSA to investigate interference of Sp1 and NF-*κ*B binding by DNA methylation are in bold and italic. The color reproduction of the figure is available at the *British Journal of Cancer* journal online.

EZ DNA Methylation-Gold Kit (Baseclear Lab Products, Leiden, the Netherlands) was used to modify 1 *μ*g of DNA. DNA methylation analysis was performed as described (van der Gun *et al*, 2010a). Bisulphite primer sequences for regions A and B are depicted in Figure 1A. The correlation between EpCAM expression and DNA methylation was assessed by Spearman's correlation test.

### Chromatin immunoprecipitation

Histone marks were determined according to the Upstate Biotechnology (Lake Placid, NY, USA) protocol and association of transcription factors was detected as described (Weinmann and Farnham, 2002) (see Supplementary Materials and Methods). Real-time PCR was performed using AbsoluteQPCR SYBRGreenROXMix (Abgene, Surrey, UK), ABI7900HT. The % input was expressed as AE^(*C*_t_input*−C*_t_ChIP)^ × Fd × 100%, where Fd is a dilution compensatory factor and AE represents the primer efficiency. Primers for regions A1, B1, B2 and C are depicted in Figure 1A and underlined in Figure 1B.

### Electromobility shift assay

OVCAR3 nuclear extract was prepared using an NE-PER kit (Pierce Biotechnology, Etten-Leur, the Netherlands). RDY681-labelled probes (Isogen, De Meern, the Netherlands) are depicted in Figure 1B. Probes were incubated with 4 *μ*g nuclear extract in 20 *μ*l binding buffer (Pierce Biotechnology) for 20′ at RT. For competition assays, a 100-fold excess of unlabelled competitor was premixed with RDY681-labelled probe and added to the binding mixture. Probes were *in vitro* methylated by M.SssI (New England Biolabs, Ipswich, MA, USA). Unmethylated probes were treated similarly but in the absence of methyl donor. Non-denaturing 4% polyacrylamide gels were visualised using Odyssey Scanner (Westburg, Leusden, the Netherlands).

## Results

### EpCAM expression in correlation with DNA methylation in ovarian cancer

**Figure 2:**
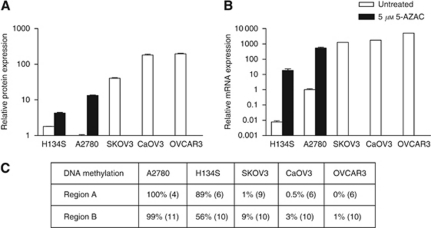
EpCAM expression in correlation with DNA methylation in ovarian cancer. To compare EpCAM expression between the different cell lines, untreated A2780 was set at 1. (**A**) The average (±s.d.) of the relative mean fluorescence intensity of one representative staining performed in triplicate is shown. (**B**) Quantitative RT–PCR analysis showing relative EpCAM mRNA levels. (**C**) The % of DNA methylation represents the number of methylated CpGs divided by the total number of CpGs present in the region. For each cell line the number of clones analysed is indicated between brackets.

Ovarian cancer cell lines were selected based on their EpCAM protein expression levels: two EpCAM-negative lines (H134S, A2780; MFI: 4.6±0.05, 2.6±0.14, respectively), SKOV3 with an intermediate EpCAM expression level (MFI: 104±3) and two cell lines (CaOV3, OVCAR3) with a high EpCAM expression level (MFI: 461±30; 496±24, respectively) (Figure 2A). The protein data are in line with the EpCAM mRNA levels (Figure 2B). To determine the role of DNA methylation in silencing EpCAM expression, the EpCAM-negative cell lines were treated with a DNA methylation inhibitor. Indeed, treatment with 5-AZAC resulted in induction of EpCAM expression in the EpCAM-negative cell lines H134S and A2780, both on protein and mRNA level (Figures 2A and B). To further investigate the correlation between EpCAM expression and DNA methylation, the methylation status of the *epcam* promoter and part of exon 1 was analysed. In the EpCAM-negative cell lines, the 61 CpGs present in region A were hypermethylated (A2780: 100±0% H134S: 89±23%), whereas region A in EpCAM-positive cell lines was hypomethylated (SKOV3: 1±3% CaOV3: 0.5±3% OVCAR3: 0±2%) (Figure 2C, Table 1). Interestingly, low to undetectable EpCAM-expressing normal epithelial ovarian cancer cells (HOSE) (Kim *et al*, 2003; Bellone *et al*, 2009) displayed a variable DNA methylation level of 15±21% (*n*=10 clones). For region B (18 CpGs), the DNA methylation levels were 99±2, 56±17, 9±13, 3±6, 1±5% for A2780, H134S, SKOV3, CaOV3, OVCAR3, respectively, (Figure 2C, Table 1). In the cell lines, an inverse correlation between EpCAM expression and DNA methylation was found (Spearman *r*=−0.97, *P*=0.02, Region A).

### Histone modifications associated with EpCAM expression

**Figure 3:**
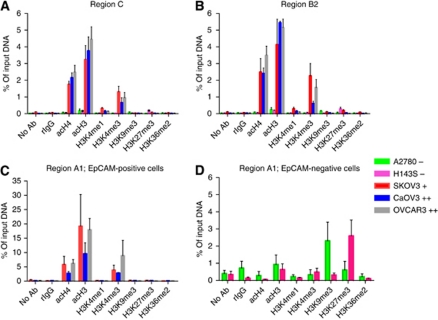
Histone modifications associated with EpCAM expression. Histone modifications associated with region C (**A**), region B2 (**B**) and A1 (**C** and **D**) within the *epcam* gene in EpCAM-negative (−) and -positive (+) cells. The absence of antibody (no Ab) and rIgG were used as negative controls. The bars represent the mean of three or more independent ChIP experiments±the s.e.m.

In EpCAM-positive cells, regions C and B2 were associated with acetylated histone 4 (acH4), acetylated histone 3 (acH3) and with trimethylation of lysine 4 of histone 3 (H3K4me3) (Figures 3A and B, Table 1). For region A1 covering the TSS, the presence of these active marks was even more pronounced (Figure 3C). In EpCAM-negative cells, association of these histone modifications was not detected, except for low levels of acH3 up to 1% of input DNA at region A1 (Figure 3D).

The repressive histone modifications H3K9me3 as well as H3K27me3 were not detected in EpCAM-positive cells. Interestingly, in the EpCAM-negative cells, region A1 was associated with repressive marks: in A2780, region A1 was associated with H3K9me3; whereas in H134S the promoter was associated with H3K27me3 (Figure 3D, Table 1).

### *In vivo epcam* gene occupancy by transcription factors

Locations of transcription factor binding sites in the *epcam* promoter as described in literature (Linnenbach *et al*, 1993; McLaughlin *et al*, 2004; Yamashita *et al*, 2007; Sankpal *et al*, 2009), as well as additional putative sites obtained by Genomatix MatInspector are shown in Figure 1B. The transcription factors screened for *in vivo* association with the *epcam* promoter were selected based on evidence for a biological role in *epcam* regulation (Gires *et al*, 2001; Tai *et al*, 2007; Yamashita *et al*, 2007; Sankpal *et al*, 2009) and their potential role in ovarian cancer (Anttila *et al*, 2000; Reimer *et al*, 2007; Min and Wei-hong, 2009; Oettgen, 2010).

**Figure 4:**
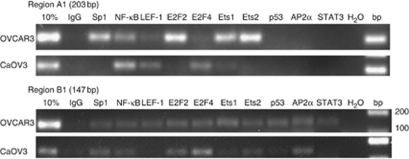
Transcription factors associated with the *epcam* gene. ChIP analysis on EpCAM-positive (OVCAR3, CaOV3) cells performed with the indicated antibodies, IgG was used as a negative control, 10%=10% of input; PCR was performed with primers for regions A1 and B1.

In the EpCAM-positive OVCAR3 cells, the promoter was associated with Sp1, NF-*κ*B, LEF-1, E2F2, Ets1 and Ets2 for both regions tested (Table 2 and Figure 4), whereas E2F4, p53, AP2*α* and STAT3 were only associated with region B1. In the EpCAM-positive CaOV3 cells, the promoter was associated with the same transcription factors as for OVCAR3, except that for p53 and STAT3 no association was detected. The transcription factors LEF-1 and Ets1 were associated with region A1, whereas association of Sp1, E2F2, Ets2 and again AP-2*α* were only found in region B1. In the EpCAM-negative cells A2780 and H134S, no association of any of the transcription factors with region A1 nor with region B1 was detected (Table 2). In addition, no association of ESE-1, SNAI1, SLUG, PEA3 and PDEF was detected neither in EpCAM-positive nor in EpCAM-negative cells (data not shown).

### Interference on binding of transcription factors by DNA methylation

The ChIP data suggest a role for NF-*κ*B and Sp1 in regulating *epcam* gene expression. Our bisulphite sequencing revealed that the CpG next to the putative binding site of NF-*κ*B (located at +27, NF-*κ*B in Figure 1B) was methylated in all clones of the *epcam*-negative cells and not methylated in the EpCAM-positive cells. Similarly, for the CpG present in a putative binding site for Sp1 (located at −32, Sp1b in Figure 1B), complete methylation in all clones was observed in the EpCAM-negative cells, whereas in the EpCAM-positive cells this particular CpG was not once methylated. Also the 2 CpGs present in two putative binding sites for Sp1 (located at −231 and −226, Sp1a in Figure 1B), were both methylated in the EpCAM-negative cells (A2780: 22/22 clones, H134S: 14/20 clones), whereas in the EpCAM-positive cells, these two CpGs were not methylated (SKOV3 and CaOV3: 1/20 clones, OVCAR3: 0/20 clones).

**Figure 5:**
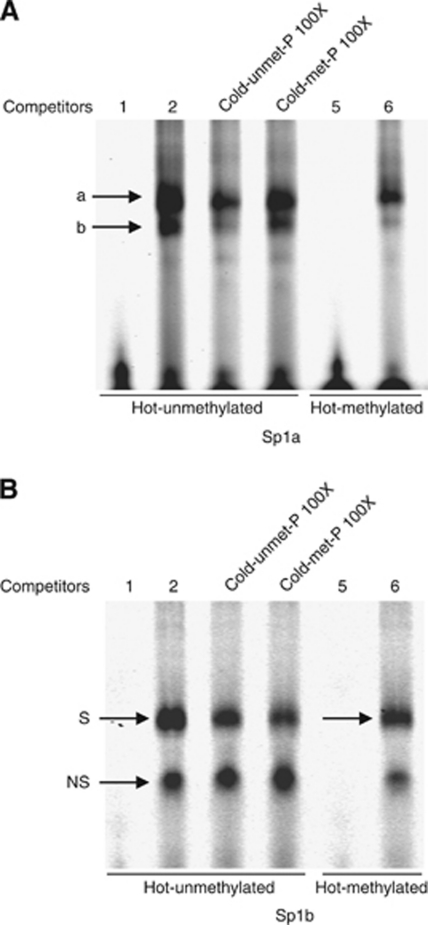
Interference of DNA methylation on binding of Sp1. Competition EMSA's were performed with hot (un)methylated (**A**) Sp1a and (**B**) Sp1b probes and nuclear extracts (NE) of OVCAR3 cells. The specificity and methylation sensitivity of the band of interest were shown by using the cold competitors (lanes 1, 5: probe; lanes 2, 6: probe with NE; lanes 3, 4: probe with NE in the presence of 100-fold excess of indicated competitor).

To investigate whether the observed DNA methylation actually interferes with binding of the transcription factors to the *epcam* promoter, EMSA competition studies were performed. Shift assay with unmethylated probe Sp1a (Figure 1B) and nuclear protein extract of OVCAR3 cells revealed two bands (a+b) (Figure 5A). Both bands were also observed for the methylated Sp1a probe, but the binding of nuclear protein to the methylated probe was less efficient than to the unmethylated probe (lane 2 compared with 6). Also competition with an excess of cold unmethylated Sp1a probe was more efficient than with a methylated probe, indicating that Sp1 binds preferentially to the unmethylated Sp1a binding site within the *epcam* promoter. Shift assay with the Sp1b probe and nuclear extract of OVCAR3 cells revealed two bands with the unmethylated probe as well as with the methylated probe (Figure 5B). One of the bands is not specific (NS), as the band intensity was not reduced with an excess of competitor. The other band indicated with an S, showed competition with both an excess of unmethylated as well as an excess of methylated probe, indicating that for this particular sequence the transcription factor binds to the Sp1b probe, regardless of DNA methylation status of the CpGs within this probe. Also for the NF-*κ*B probe, no difference in binding patterns to the methylated and unmethylated NF-*κ*B probes was observed (data not shown).

## Discussion

Epigenetic aberrations, including DNA methylation and histone modifications, are well established in the development and progression of ovarian cancer (Barton *et al*, 2008; Balch *et al*, 2009). A number of protein-coding genes are overexpressed in ovarian cancer because of loss of DNA methylation, including maspin, claudin-3 (Honda *et al*, 2007) and claudin-4 (Barton *et al*, 2008). In addition, overall loss of the repressive histone mark H3K27me3 has been associated with poor prognosis in ovarian cancer (Wei *et al*, 2008). In this study, we set out to unravel the epigenetic marks underlying EpCAM overexpression in ovarian cancer.

In our ovarian cancer cell line panel, EpCAM expression was inversely correlated with the DNA methylation status of the promoter and part of exon 1, as reported for several other tumour types (Spizzo *et al*, 2007; Tai *et al*, 2007; Yu *et al*, 2008; van der Gun *et al*, 2008). Interestingly, treatment of our EpCAM-negative ovarian cancer cells with a DNA methylation inhibitor induced EpCAM expression, both on mRNA and protein level. The role of DNA methylation in silencing *epcam* has been previously published by us for the intermediate *epcam* expressing SKOV3 ovarian cancer cells, and is in line with observations in other tumour types (Spizzo *et al*, 2007; Tai *et al*, 2007; van der Gun *et al*, 2008). In normal cells (HOSE), we did not find DNA hypermethylation, even though in several ovarian cancer cell lines, including SKOV3, CaOV3 and OVCAR3, EpCAM mRNA was reported to be 3 log higher compared with HOSE cells (Kim *et al*, 2003). Also on protein level, HOSE cultures showed negative to negligible levels of EpCAM expression (Bellone *et al*, 2009). This unexpected low DNA methylation level for EpCAM in HOSE cells is in line with data of differentiating human embryonic stem cells, where *epcam* silencing was not associated with increased DNA methylation (Lu *et al*, 2010). In these cells, *epcam* silencing was associated with a reduction of active histone marks and an enhancement of repressive histone marks (Lu *et al*, 2010). Also in our panel of EpCAM-negative cell lines, we found the silenced *epcam* promoter to be associated with repressive marks (H3K9 or H3K27 trimethylation), and relative low levels of active histone marks (H3/H4 acetylation, H3K4 trimethylation) were observed.

The epigenetic marks found in the EpCAM-negative cells indicate a closed chromatin conformation, which might explain the absence of association of any of the tested transcription factors with the *epcam* gene in H134S and A2780 cells. Out of the 16 tested transcription factors, 10 were associated with the promoter in the EpCAM-expressing cells. We are the first to show association of AP2*α*, Ets1, Ets2, E2F2, E2F4 and STAT3 with the *epcam* gene. Of special interest are the associations found for nuclear AP2*α* and Ets1, as high levels of these transcription factors have been related to poor prognosis in ovarian cancer (Anttila *et al*, 2000; Oettgen, 2010). Similarly, a high E2F2 to E2F4 ratio was reported to be of prognostic value for ovarian cancer-free survival (Reimer *et al*, 2007). Also STAT3 is overexpressed in ovarian cancer compared with normal or benign ovarian tumour tissue, and its expression was significantly higher in FIGO stage III/IV compared with stage I/II (Min and Wei-hong, 2009). Although our data indicate that the relation between transcription factors and clinical parameters might partially take place via EpCAM expression regulation, the direct biological significance needs to be further established.

For the transcription factors, p53, NF-*κ*B, Sp1 and LEF-1 (Yamashita *et al*, 2007), evidence for regulating EpCAM expression was previously demonstrated in other tumour types. Although wild-type p53, but not mutant p53, has been reported to repress EpCAM expression (Sankpal *et al*, 2009), we did not observe p53 association in EpCAM-negative cells. The observed association of p53 in the EpCAM-positive, p53-mutant OVCAR3 cells (Kolfschoten *et al*, 2002) is in agreement with the acetylated histones associated with the promoter in these cells, as mutant p53 recruits the histone acetyl transferase p300 (Strano *et al*, 2007). In the presence of p300, the repressive action of NF-*κ*B on *epcam* promoter activity was abolished (Gires *et al*, 2001), which might explain the association of NF-*κ*B and acetylated histones with the EpCAM promoter in EpCAM-positive cells. Also Sp1 has previously been reported to be involved in EpCAM transcription (Tai *et al*, 2007). Interestingly, our observation that the CpGs located around −230 within the Sp1 binding sites were methylated in EpCAM-negative ovarian cell lines and unmethylated in the EpCAM-positive lines was also reported for several other types of tumours (Yu *et al*, 2008). Together with our *in vitro* finding that methylation of these particular CpGs affects Sp1 binding, this region is currently explored by us for targeted DNA methylation approaches (van der Gun *et al*, 2010a).

Apart from DNA methylation and histone modifications, other epigenetic mechanisms, including non-coding RNAs, may be (directly and indirectly) involved in *epcam* gene regulation. In this respect, microRNA-181 has been shown to upregulate *epcam* gene expression, possibly via a positive feedback loop between miR-181 and Wnt/*β*-catenin signalling (Ji *et al*, 2011). These observations are in line with our data showing association of the Wnt-pathway transcription factor LEF-1 with the active *epcam* promoter. Alternatively, several endogenous non-coding RNAs have been reported to be capable of modulating gene expression directly on the transcriptional level, for example, by inducing DNA methylation (Morris, 2011).

At present, EpCAM is exploited as therapeutic target in several antibody-based clinical trials. Recently, the European Medicines Agency approved the use of catumaxomab for the intraperitoneal treatment of malignant ascites (Bokemeyer, 2010). The oncogenic role of EpCAM broadens the interest to use EpCAM not only as an immunotarget but also as a target for epigenetic silencing. In this respect, transient siRNA-mediated silencing of EpCAM expression has been shown to reduce the oncogenic potential of breast (Osta *et al*, 2004), gastric (Du *et al*, 2009), hepatocellular (Yamashita *et al*, 2009) and oral squamous cell (Yanamoto *et al*, 2007) carcinomas.

To silence gene expression in a more sustained way, targeted DNA methylation has been achieved by fusing a DNA methyltransferase to a DNA binding domain like zinc-fingers (Smith *et al*, 2008). Similarly, transcription effector domains fused to zinc-fingers targeting the *epcam* promoter modulated *epcam* promoter activity (Gommans *et al*, 2007). Recently, we showed that an EpCAM-specific triple helix-forming oligonucleotide coupled to a methyltransferase variant is able to target methylation predominantly to a specific CpG in the *epcam* promoter (van der Gun *et al*, 2010a). Interestingly, targeted methylation in living cells induced dense methylation up to 380 bp on both sides of the target site (Li *et al*, 2007), suggesting that initial DNA methylation might serve as a trigger for DNA methylation spreading. Elucidating the regulation mechanisms of *epcam* in ovarian cancer as presented here thus opens up new possibilities to exploit EpCAM as a therapeutic target.

## Figures and Tables

**Table 1:** Epigenetic marks associated with EpCAM expression

	**A2780**	**H134S**	**SKOV3**	**CaOV3**	**OVCAR3**
EpCAM expression	−	−	+	++	++
					
*Active histone marks, region C/B2*					
acH4	−	−	+	+	+
acH3	−	−	+	+	+
H3K4me3	−	−	+	+	+
					
*Active histone marks, region A1*					
acH4	−	−	++	++	++
acH3	−	−	++	++	++
H3K4me3	−	−	++	++	++
					
*Repressive histone marks, region C/B2*					
H3K9me3	−	−	−	−	−
H3K27me3	−	−	−	−	−
					
*Repressive histone marks, region A1*					−
H3K9me3	+	−	−	−	−
H3K27me3	−	+	−	−	−
					
DNA methylation Region B/A	+++	++	+/−	−	−

**Table 2:** Transcription factors associated with the *epcam* gene (+=association, −=no association)

	**Sp1**	**NF-*κ*B**	**LEF-1**	**E2F2**	**E2F4**	**Ets1**	**Ets2**	**p53**	**AP2*α***	**STAT3**
*OVCAR3*										
A1	+	+	+	+	−	+	+	−	−	−
B1	+	+	+	+	+	+	+	+	+	+
										
*CaOV3*										
A1	−	+	+	−	+	+	−	−	−	−
B1	+	+	−	+	+	−	+	−	+	−
										
*A2780*										
A1	−	−	−	−	−	−	−	−	−	−
B1	−	−	−	−	−	−	−	−	−	−
										
*H134S*										
A1	−	−	−	−	−	−	−	−	−	−
B1	−	−	−	−	−	−	−	−	−	−
