# Supplementary material for: Transcription factors and molecular epigenetic marks underlying EpCAM overexpression in ovarian cancer
Source: Br J Cancer. 2011 Jun 21;105(2):312–9. doi: 10.1038/bjc.2011.231 (PMC3142811; doi:10.1038/bjc.2011.231)
Supplement: Supplementary Information [file bjc2011231x1.doc]

**Supplementary online material**

**Materials and Methods**

**Chromatin ImmunoPrecipitation** Histone modifications were determined using antibodies from Upstate Biotechnology (Lake Placid, NY, USA): rabbit IgG, acH4, acH3, H3K4me1, H3K4me3, H3K9me3, H3K27me3, H3K36me2 according to the Upstate protocol with the following modifications. Fixated cells were sonicated using a Bioruptor (High, 15 cycles: 30’’on 30’’off) (Diagnode, Liège, Belgium). Chromatin fragments were diluted 2.5-fold and precleared for 2h at 4°C. Incubation with antibody was followed by 2h incubation with 60 μl protein A/G-agarose beads. DNA was purified using QiaQuick DNA spin columns (Qiagen, Venlo, Netherlands). To detect association of transcription factors, ChIP was performed as described (Weinmann & Farnham, 2002), using the antibodies: mouse IgG, LEF-1(REMB6)TCF (Millipore, Amsterdam, Netherlands), Sp1 (Upstate), STAT3 (Upstate), and from Santa Cruz Biotechnology (Heidelberg, Germany): NF-κBp50(NLS), NF-κBp65(A), ESE-1(H-270), SNAI1(E-130), SLUG(H-140), Ets-1(C-20), Ets-2(C-20), AP2-α(C-18), PEA3(H-120), PDEF(H-250), E2F-2(C-20), E2F-4(C-20) and p53. For the Real Time PCR a freshly made calibration line was included for every primer set used and PCR was finished with a dissociation curve. Conventional PCR was performed according to the protocol of Fermentas (St. Leon-Rot, Germany).
